# The influence of prolonged instrument manipulation on gas leakage through trocars

**DOI:** 10.1007/s00464-023-10240-5

**Published:** 2023-07-13

**Authors:** Daniel Robertson, Matthijs van Duijn, Alberto Arezzo, Yoav Mintz, Technology Committee of the EAES, Luigi Boni, Ludovica Baldari, Thomas Carus, Manish Chand, Hans Fuchs, Fanny Ficuciello, Stefania Marconi, George Mylonas, Young Woo Kim, Kiyokazu Nakajima, Marlies Schijven, Pietro Valdastri, Chen Sagiv, Pietro Mascagni, Piotr Myśliwiec, Wanda Petz, Francisco Sánchez-Margallo, Tim Horeman-Franse

**Affiliations:** 1 Department of Biomechanical Engineering, Faculty of Mechanical Engineering, Delft University of Technology, Mekelweg 2, 2628 CD Delft, The Netherlands; 2 Department of Surgical Sciences, University of Torino, Turin, Italy; 3 Department of General Surgery, Hadassah Hebrew University Medical Center, Jerusalem, Israel

**Keywords:** Trocar leakage, Trocar manipulation, Laparoscopic model

## Abstract

**Background:**

During laparoscopic surgery, CO_2_ insufflation gas could leak from the intra-abdominal cavity into the operating theater. Medical staff could therefore be exposed to hazardous substances present in leaked gas. Although previous studies have shown that leakage through trocars is a contributing factor, trocar performance over longer periods remains unclear. This study investigates the influence of prolonged instrument manipulation on gas leakage through trocars.

**Methods:**

Twenty-five trocars with diameters ranging from 10 to 15 mm were included in the study. An experimental model was developed to facilitate instrument manipulation in a trocar under loading. The trocar was mounted to a custom airtight container insufflated with CO_2_ to a pressure of 15 mmHg, similar to clinical practice. A linear stage was used for prolonged instrument manipulation. At the same time, a fixed load was applied radially to the trocar cannula to mimic the reaction force of the abdominal wall. Gas leakage was measured before, after, and during instrument manipulation.

**Results:**

After instrument manipulation, leakage rates per trocar varied between 0.0 and 5.58 L/min. No large differences were found between leakage rates before and after prolonged manipulation in static and dynamic measurements. However, the prolonged instrument manipulation did cause visible damage to two trocars and revealed unintended leakage pathways in others that can be related to production flaws.

**Conclusion:**

Prolonged instrument manipulation did not increase gas leakage rates through trocars, despite damage to some individual trocars. Nevertheless, gas leakage through trocars occurs and is caused by different trocar-specific mechanisms and design issues.

**Supplementary Information:**

The online version contains supplementary material available at 10.1007/s00464-023-10240-5.

During minimally invasive surgery (MIS) like laparoscopic surgery, the surgical site is inflated with carbon dioxide (CO_2_) to create sufficient space for tissue manipulation. An insufflator inflates CO_2_ gas to differential pressures, commonly between 8 and 12 mmHg [1, 2]. Trocars act as the access port between the operating room and intra-abdominal environments. Valves inside the trocar prevent CO_2_ from escaping the intra-abdominal cavity. Despite the presence of trocar valves, recent studies show that gas leakage could still occur [3, 4].

Leaked gas could contain carcinogenic particles, viruses, and other ultrafine particles, exposing operating personnel [5–9]. Aerosolized human papillomavirus (HPV) and hepatitis B virus have already been detected in surgical smoke [10]. Additionally, SARS-CoV-2 is known to be viable in aerosols for multiple hours, creating a theoretical risk of transmission [11]. Surgeons expressed concern about contracting COVID-19 during surgery, stressing the risks in clinical practice [12], resulting in a growing need for research clarifying the safety of gas leakage during MIS. Previous research addressing gas leakage has mainly focused on investigating the composition of intra-abdominal gas and its harmful effects [5, 13–15].

Literature describes three different leakage methods how contaminated gas can leak into the operating room: leakage through the instrument shaft, between the trocar and the incision, and through the trocar [3]. Leakage through the instrument’s shaft depends on the individual instrument design and can be considered constant during surgery. Leakage between the trocar and the incision is associated with incision size, abdominal wall composition, and entry technique, making leakage quantities partly subject to the personal skill level of the surgeon. Finally, trocar leakage is influenced by the interaction between the trocar and the instrument [4].

There have been studies that investigated this interaction between the trocar and instrument; however, these do not evaluate whether leakage increases over the longer period of a surgical procedure. Literature has shown that especially trocars that can facilitate instruments with diameters of 10 mm and above show significant gas leakage [3, 4, 16]. Therefore, this study investigates the influence of prolonged instrument manipulation on gas leakage through trocars with internal diameters of 10 mm and above. Since such loading is representative of clinical practice, insight into the trocars’ performance under these circumstances is paramount in illuminating gas leakage during surgery.

## Methods

### Protocol

The instrument manipulations performed during laparoscopic surgery combine axial, pivotal, and radial displacements. To simulate the influence of instrument manipulation during clinical practice on gas leakage through trocars, a protocol was developed to measure the difference in gas leakage before and after manipulation. As the study did not involve human participants or animals, no approval of the Institutional Review Board (IRB) was required.

Static measurements were taken before (steps 1, 2, 3) dynamic measurements as a baseline and after as control measurements (steps 7, 8, 9) to measure the difference in gas leakage after manipulation. The steps of the protocol are described in Table 1. The order of the steps was chosen to increase wear over time. Per trocar, two test rounds of 9 steps were performed, first using a 5-mm instrument and then using a 10-mm instrument. During the first and last steps of the protocol, the trocar was empty where step 9- of the 5-mm instrument sequence doubled as step 1 of the 10-mm instrument sequence. The inserted instrument in the trocar was either moving in the axial direction or static and was either loaded or unloaded.

**Table 1:**
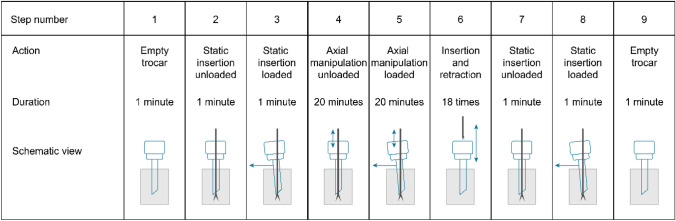
Schematic overview of measurements

When the instrument was used, it was inserted so that the tip passed all trocar valves and protruded from the cannula. The loaded steps were performed with a radial load connected to the trocar to mimic the force between the instrument and the trocar. A static weight of 1100 g was attached to the trocar using a wire and pulley, resulting in a moment of 0.54 nm, this is in line with loads applied during laparoscopic surgery [17, 18].

The dynamic steps included unloaded and loaded axial manipulation for 20 min, with a stroke length of 30 mm and a velocity of 25 mm/s. This is a common velocity during instrument manipulation by experienced laparoscopic surgeons [19]. Step 6 simulates instrument exchanges by consecutively inserting and entirely removing the instrument past all trocar valves 18 times. The removal and insertion velocity were set to 40 mm/s. This is the average instrument exchange rate calculated during 40 min of surgery [20–30].

Before the measurement of every trocar, the container was flushed with 35 L of CO_2_ to ensure saturation with CO_2_. Subsequently, the silicone nozzle was sealed, and the container was pressurized to 15 mmHg with a flow of 15 L/min to obtain a zero leakage flow measurement for 30 s to calibrate the offset of the flow sensor. The test sequence was initiated with the obturator inside the trocar to mimic clinical practice. Measurements were timed, started, and stopped manually using a custom interface on the computer.

All trocar valves were inspected for visual damage after the final measurement. If leakage was suspected, a soap bubble test was performed to locate the leakage. Additionally, trocar-specific testing was conducted based on observations during testing (e.g., peaks in the flow curve, audible gas leakage, visible valve damage) to determine the cause of the leakage.

### Materials

#### Trocars

Surgeons associated with the European Association for Endoscopic Surgery (EAES) were asked to send in trocars they would use in clinical practice. Trocars with a nominal inner diameter of 10 mm or larger were included. Both new disposable and reusable trocars with new valves were included. All trocars were inspected for defects and categorized prior to testing.

#### Experimental setup

**Fig. 1:**
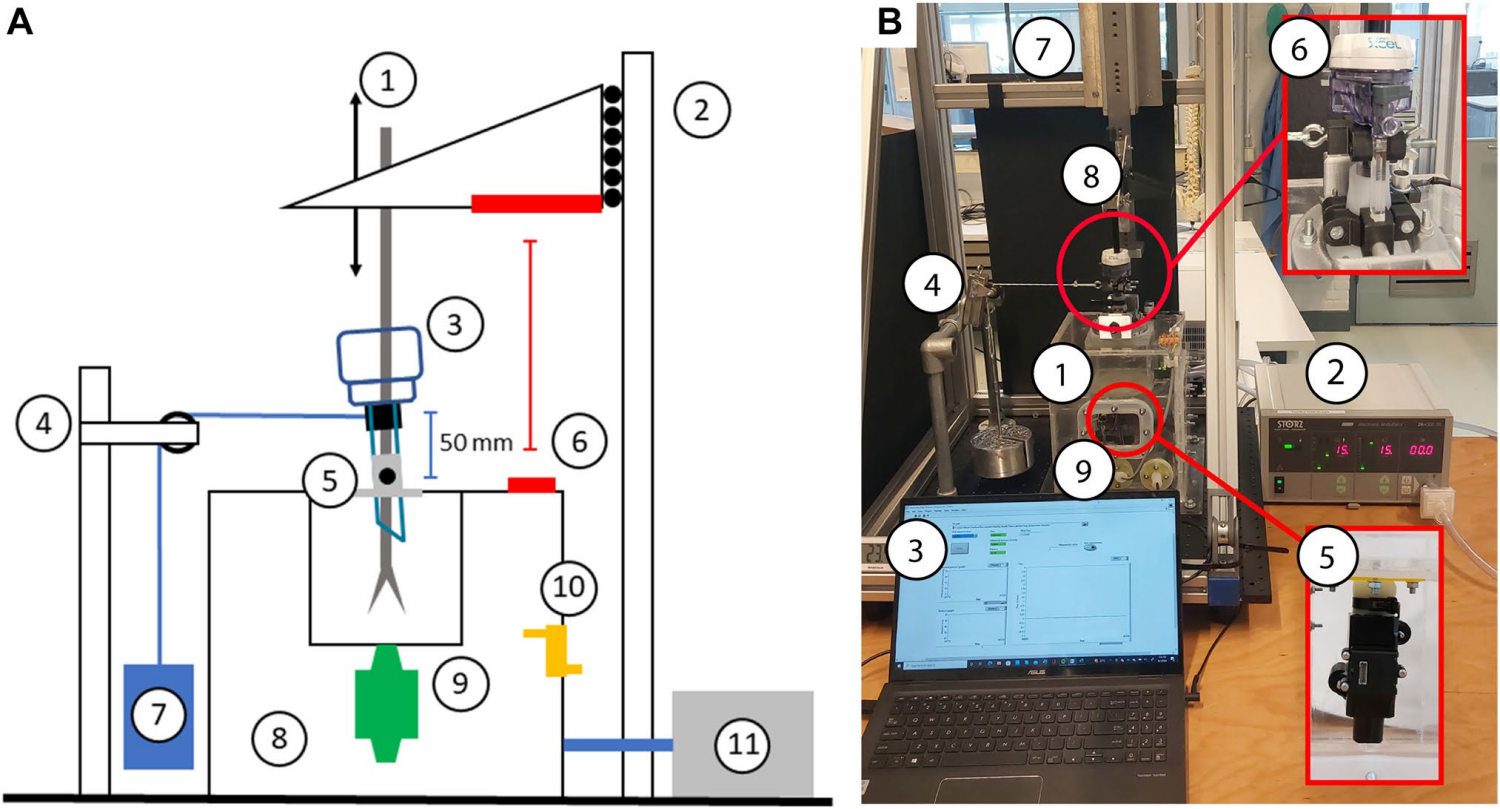
**A** Schematic of the experimental setup. 1: Laparoscopic instrument, 2: linear stage, 3: trocar, 4: pulley frame, 5: trocar Mount, 6: distance sensor, 7: weight, 8: airtight container, 9: flow sensor, 10: differential pressure sensor, 11: insufflator. **B** Overview test setup. 1: acrylic container, 2: insufflator, 3: laptop running LabView, 4: static load, 5: flow sensor, 6: trocar mount, 7: linear stage, 8: laparoscopic instrument, 9: Arduino UNO R3 microcontroller

A schematic overview of the experimental setup is presented in Fig. 1. An airtight, transparent, rigid acrylic container (200 × 200 × 250 mm^3^) with a wall thickness of 5 mm was manufactured, with multiple through holes for the trocar, insufflation hose, pressure sensor outlet, and wiring. The through holes were air tightened with custom silicone nozzles. The silicone nozzle for the trocar had a smaller diameter than the trocar to prevent leakage. The trocar’s pivot point was fixed, and a second clamp was connected to the trocar 50 mm above the pivot point to attach the weight for the loaded steps. Two lids allowed access to the electronics.

Laparoscopic instruments were used to ensure realistic friction between the instrument and the trocar valve. The instrument handle was removed, and the proximal end of the shaft was capped to prevent leakage through the instrument shaft. All tests were conducted with a 5-mm LigaSure Blunt Tip Laparoscopic Sealer (Covidien, Dublin, Ireland) and a 10-mm Endo Babcock instrument (Covidien, Dublin, Ireland). No additional lubrication was added during testing, following the instructions for use provided with new trocars.

The instrument was connected to a linear stage (Festo EGSL-BS-55-250-12.7P, Festo, Esslingen am Neckar, Germany) that ensures only axial displacement of the instrument. The Festo Configuration Tool (Festo, Esslingen am Neckar, Germany) was used to pre-program the displacements and velocities.

During the experiment, the container was pressurized using an insufflator (Electronic Endoflator Model 26 4305 20, Karl Storz, Tuttlingen, Germany) connected to a CO_2_ bottle. To prevent the insufflation flow from distorting the opposing leakage flow, the insufflation hose was connected to a dedicated nozzle at the bottom of the container, instead of to the trocar.

#### Data acquisition and processing

A flow sensor (Honeywell Zephyr, HAFUHH0050L4AXT, Honeywell International Inc., Charlotte, North Carolina, USA) was located collinearly to the trocar to measure gas leakage. The flow sensor was calibrated with CO_2_ using a 3-L calibration syringe (Hans Rudolph series 5530, Hans Rudolph Inc., Shawnee, Kansas, USA). The differential pressure between the lab environment and the inside of the container was measured using a pressure sensor (Honeywell ABPMRRN060MGAA5, Honeywell International Inc., Charlotte, North Carolina, USA). The distance between the instrument mount and container was measured using an ultrasonic distance sensor (HC-SR04, SparkFun Electronics, Niwot, Colorado, USA) to track instrument displacement during instrument manipulation. CO_2_ concentration levels were monitored using a thermal conductivity sensor (Sensirion STC31, Sensirion AG, Stäfa, Switzerland) and were displayed using an Arduino Uno R3.

Sensor data were retrieved with an Arduino Uno R3 microcontroller with a sampling frequency of 40 Hz. Flow, pressure, and distance data from the microcontroller were recorded in LabVIEW (Version 18.0f2, National Instruments, Austin, Texas, USA).

Diadem (Version 22.0.0f8498, National Instruments, Austin, Texas, USA) was used for data processing, and OriginPro (Version 9.8.0.200, OriginLab Corporation, Northampton, Massachusetts, USA) was used for visualizations. The flow data per step was summarized as the median value. The difference between the control and baseline measurement was presented by subtracting the baseline from the control measurement. Also, the difference between leakage at the start and end of a dynamic measurement was compared. Absolute leakages larger than 0.25 L/min were reported, and leakages larger than 0.1 L/min were reported for the difference between the control and baseline.

## Results

### Included trocars

A total of 25 trocars from six different brands were included. Twelve unique trocar types were available, with diameters ranging from 10 to 15 mm, and 22 trocars were disposable. Equal trocar types were grouped. An overview of all included trocars is presented in Table 2. One reusable trocar type was included with a reusable lower valve and a disposable upper valve. This was listed as three separate trocars as the top valve was replaced after each use. This reusable trocar was only compatible with 10-mm instruments. Therefore, the three trocars from group 5 were tested with 10-mm instruments, and the 22 other trocars were tested with both 5-mm and 10-mm instruments. None of the trocars showed visible defects before testing.

**Table 2:** Overview of included trocars

Label	Group	Brand	Type	Use type	Diameter [mm]	Reference
1–3	1	Sejong Medical	Laport Disposable Trocar System	Disposable	12	T11-1210
4–6	2	Applied Medical	Kii Fios First Entry	Disposable	12	CFF73
7–9	3	Applied Medical	Kii Optical Access System	Disposable	15	C0R37
10–12	4	Covidien	VersaStep Plus	Disposable	12	VS101012P
13–15	5	Karl Storz	HICAP w/multifunctional valve	Reusable	12	30107
16–17	6	Ethicon	Endopath Excel	Disposable	12	B12LT
18–19	7	Ethicon	Endopath Excel	Disposable	11	B11LT
20–21	8	Ethicon	Endopath Excel	Disposable	12	D12LT
22	9	Covidien	VersaOne	Disposable	12	NONB12STF
23	10	Covidien	VersaPort Plus V2	Disposable	12	179096PF
24	11	Covidien	Auto Suture	Disposable	10	OMS-T10BT
25	12	Mölnlycke Health Care	Bladeless Dilating Tip	Disposable	12	899312

### Raw data

**Fig. 2:**
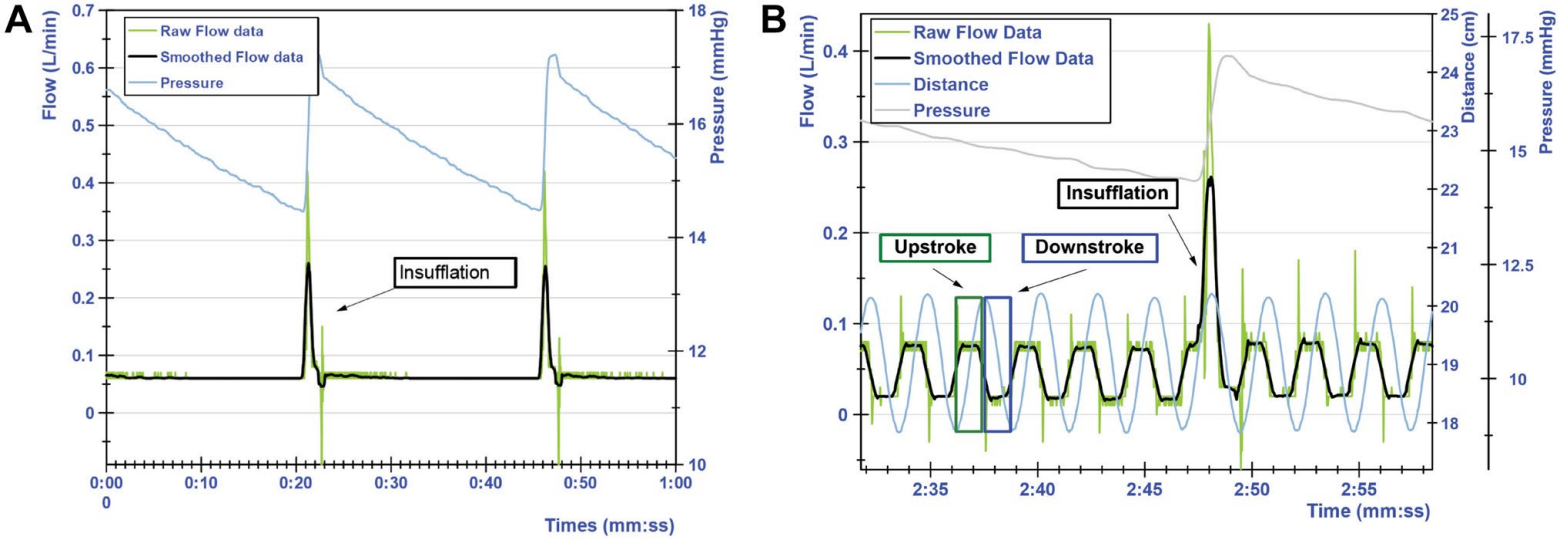
Visualization of the raw, smoothed flows, and pressure inside the trocar. **A** Example of static flow measurement. **B** Dynamic flow measurement

Figure 2a shows an example of a one-minute unloaded static measurement with a 5-mm instrument. The flow remains steady at around 0.06 L/min. The two peaks in the flow data occurred are caused by insufflation and correspond to peaks in the pressure curve. Figure 2b shows a 30-s sample of a 20-min dynamic unloaded measurement with a 5-mm instrument. The distance curve oscillates corresponding to the up and down stroke of the instrument. One additional peak around 0.4 L/min occurs during insufflation.

### Manipulation trocar leakage

**Fig. 3:**
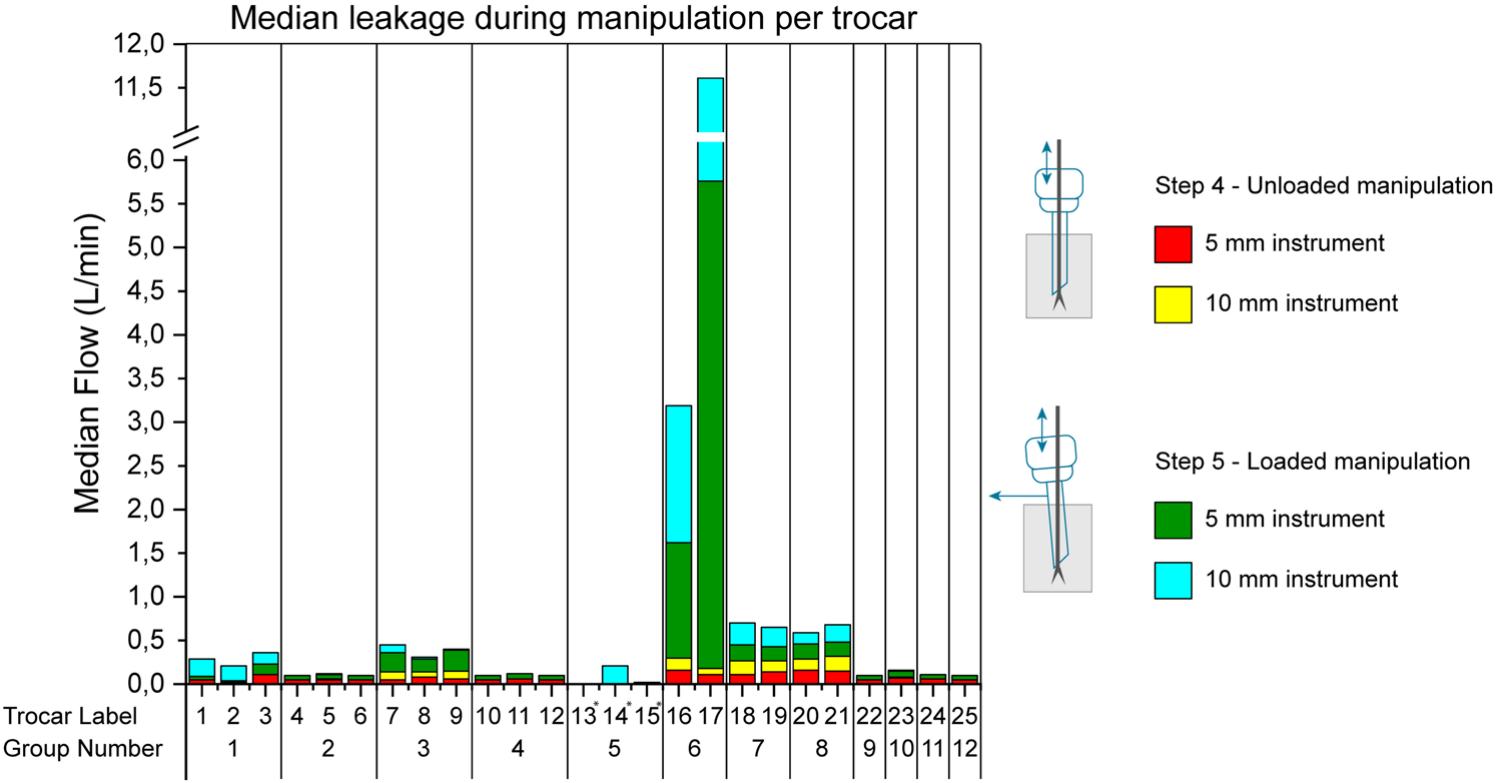
Median leakage during manipulation per trocar. Trocar numbers indicated with * were only tested with a 10-mm instrument

The rate of gas leakage from the trocars during manipulation (steps 4 and 5) is shown in Fig. 3. The 25 trocars underwent four measurements, except in group 5, as they could only be tested with a 10-mm instrument. Only group 6 showed leakage rates higher than 0.25 L/min which occurred during loaded manipulation. For trocar 17, leakage rates were 5.58 L/min and 5.85 L/min for loaded manipulation with a 5-mm and 10-mm instrument, respectively.

**Fig. 4:**
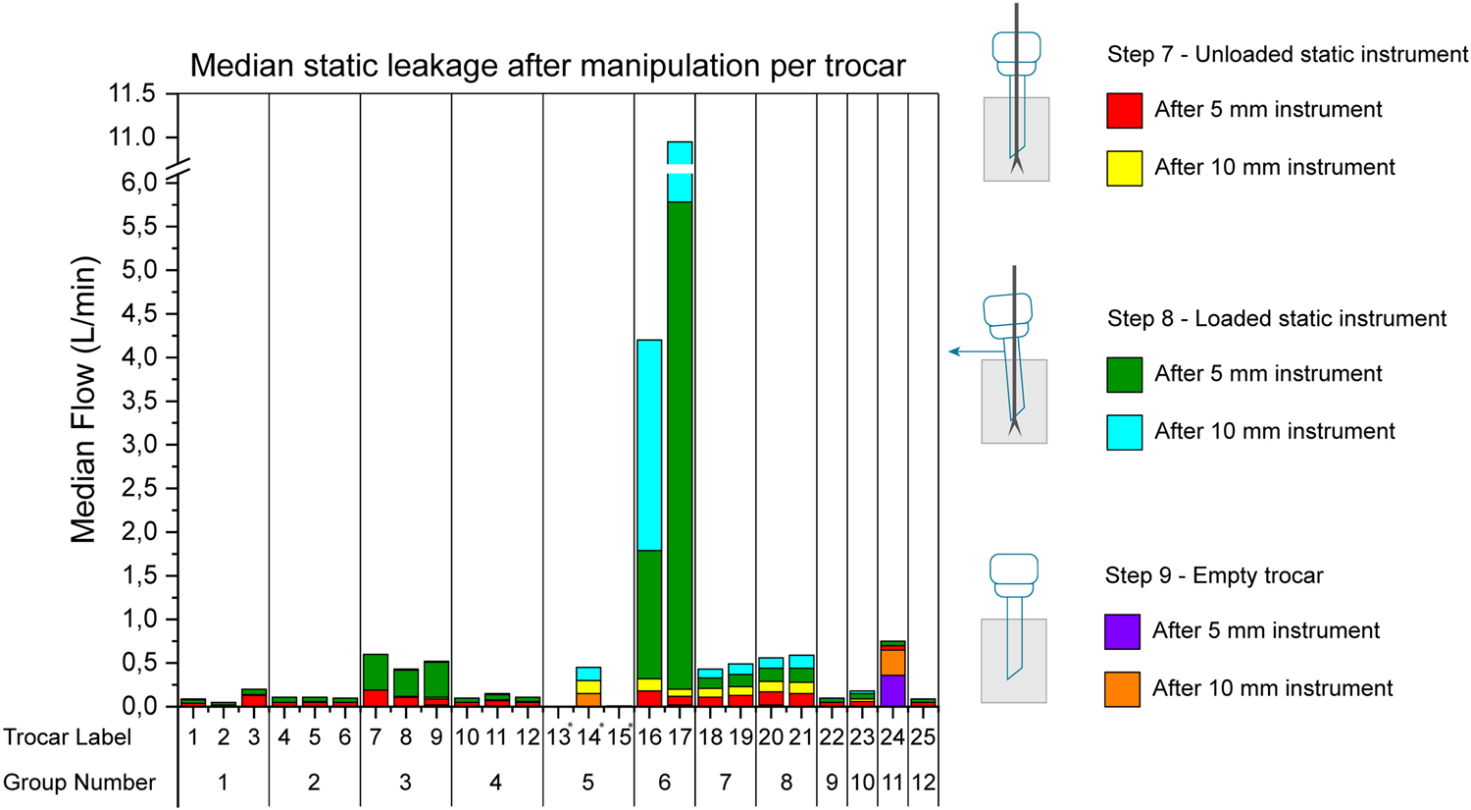
Median leakage during manipulation per trocar. Trocar numbers indicated with * were only tested with a 10-mm instrument

Median leakage rates after manipulation (steps 7, 8, 9) per trocar are shown in Fig. 4. Six measurements are presented for all trocars, while only three are for group 5. Of 141 measurements, 9 (7%) had a median leakage greater than 0.25 L/min.

Trocars in group 3 showed a median leakage ranging from 0.30 to 0.41 L/min for loaded measurements with a 5-mm instrument. Trocars in group 6 showed the highest leakage rates, where trocar 17 had a median leakage for the loaded measurements at step 8 of 5.58 L/min and 5.17 L/min for the 5- and 10-mm instruments, respectively. Trocar 24 showed median empty trocar leakage after the 5-mm and 10-mm instruments, respectively, of 0.29 and 0.36 L/min.

**Fig. 5:**
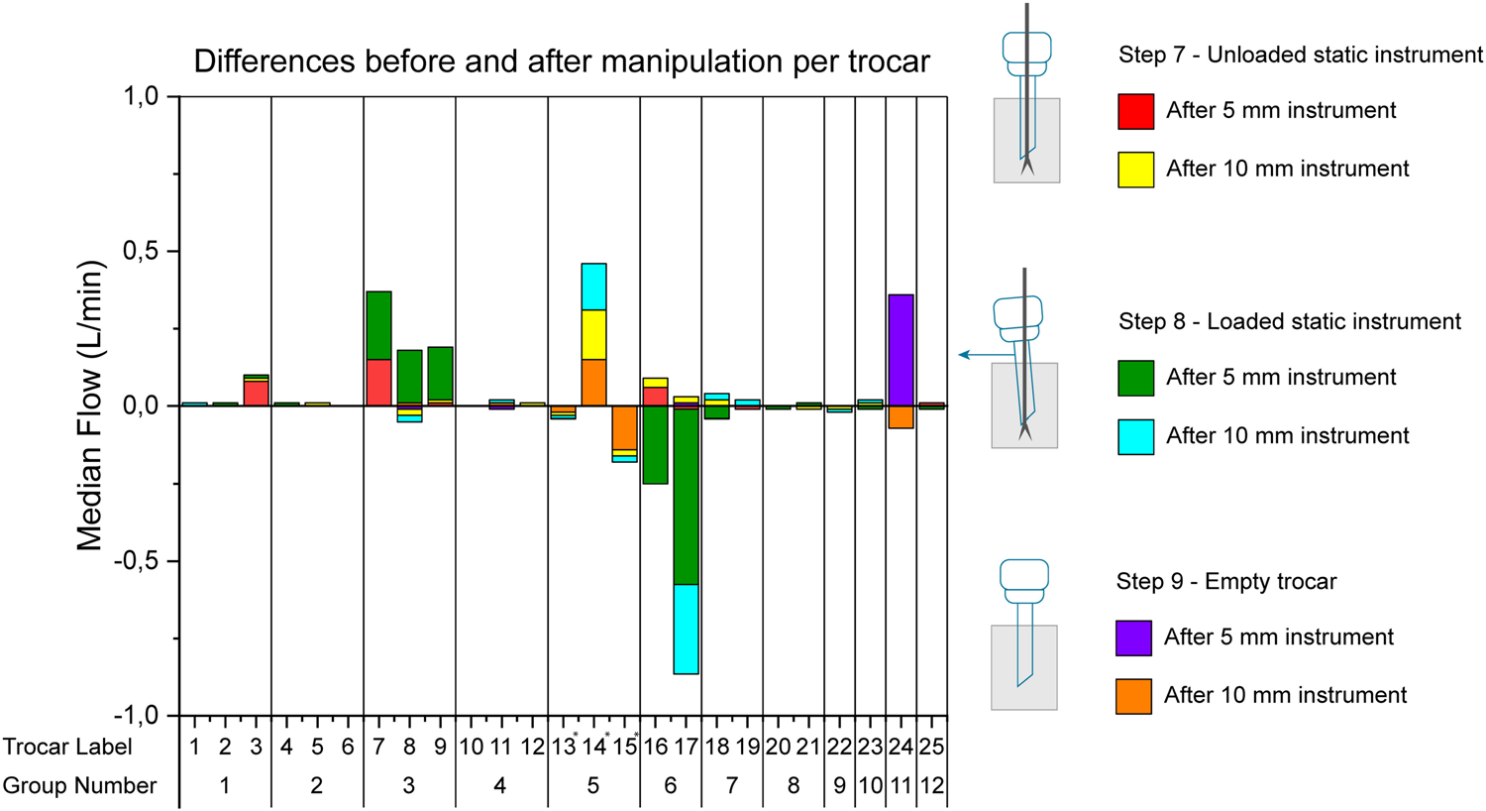
Median differences before and after manipulation per trocar calculated by subtracting the static baseline measurement from the static control measurement. Trocar numbers with * indication were only tested with a 10-mm instrument

Figure 5 shows the differences between the static measurement after and before. Positive values indicate that median leakage was higher after manipulation. An increase in median leakage larger than 0.1 L/min was seen in trocars 7, 8, 9, 14, and 24.

All trocars from group 3 (trocars 7, 8, 9) showed an increase in leakage after manipulation ranging from 0.17 to 0.22 L/min for the loaded trocar inserted with a 5-mm instrument, increasing from 0.19 to 0.39 L/min.

Trocar 14 from group 5 showed an increase in leakage for both unloaded and loaded measurements with a 10-mm instrument. Baseline leakage for the unloaded and loaded control measurements was − 0.01 L/min and 0.0 L/min compared to 0.15 L/min and 0.15 L/min for the 5- and 10-mm instruments, respectively. Leakage of the empty trocar measurement also increased after manipulation from 0.0 to 0.15 L/min.

Trocars 15, 16, and 17 show a reduction in leakage greater than 0.1 L/min after manipulation. Trocar 15 showed a reduction of 0.14 L/min in median leakage at step 9 with a 10-mm instrument. Trocars 16 and 17 of group 6 showed reduced leakage rates ranging from 0.25 to 0.56 L/min after manipulation in loaded measurements. Lastly, trocar 24 showed an increased median leakage of 0.36 L/min in the empty trocar measurement after manipulation with a 5-mm instrument. Additionally, trocar 17 also showed a reduction at step 8 for the 10-mm instrument of 0.29 L/min.

### Trocar inspection

**Fig. 6:**
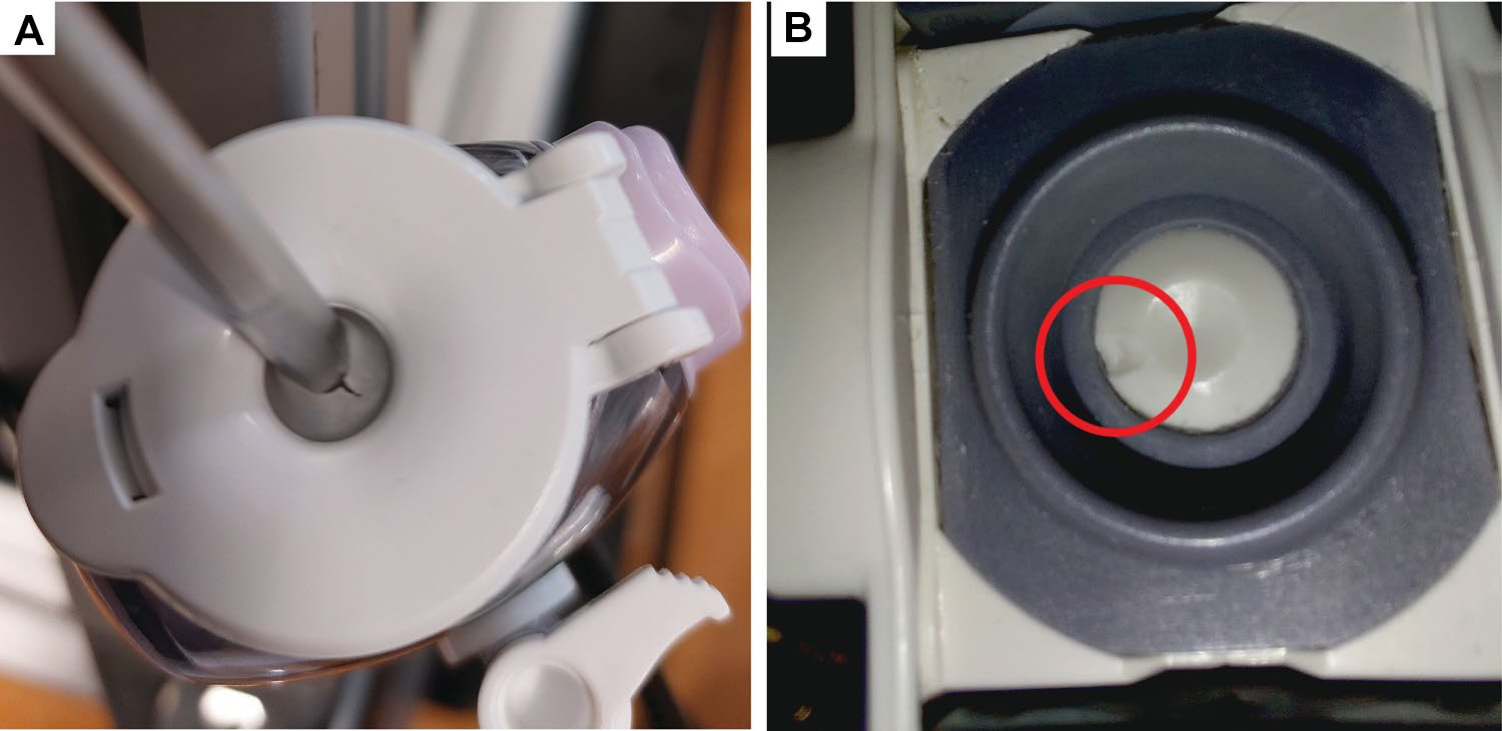
Observed damage to trocars after inspection. **A** Tear in the upper valve of trocar 1. **B** Visible damage to the lower valve of trocar 24

Of all 25 trocars, trocars 1 and 24 showed visible damage after the measurements. In trocar 1, a tear was found in the upper valve (Fig. 6a). This was caused by the upper trocar valve being clamped between the instrument and the trocar during loaded manipulation with a 5 mm instrument. A video of the soap bubble test performed on trocar 1 was added as a supplemental file.

Trocar 24 showed valve damage to the lower valve after manipulation (Fig. 6b). Before valve damage, the empty trocar leakage was 0.0 L/min. After manipulation, the damaged valve caused a median leakage of 0.36 L/min.

**Fig. 7:**
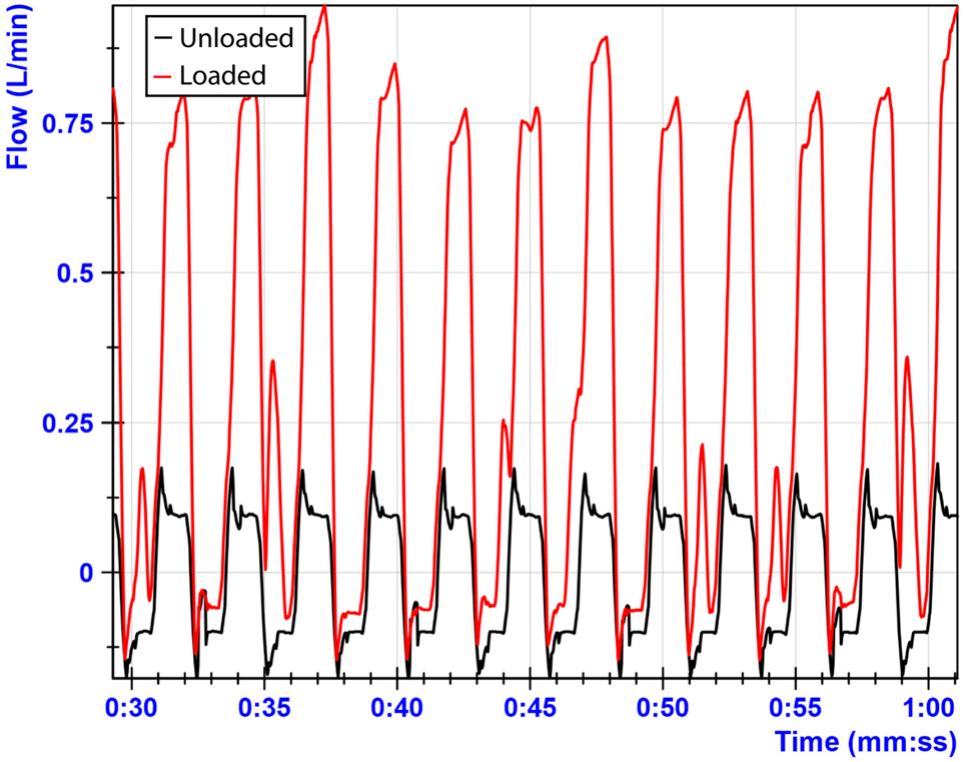
Comparison of unloaded versus loaded manipulation of trocar 1

All trocars from group 1 had audible leakage during the upstroke of the loaded dynamic measurement with a 10-mm instrument and were further investigated. Figure 7 shows a sample of both the unloaded and loaded leakage curve for manipulation with a 10-mm instrument. This pattern was consistent during the full 20 min of measurement data. The figure shows that leakage rates of the loaded measurement are larger during the upstroke than the unloaded measurement. For the down stroke, both curves show similar leakage rates.

**Fig. 8:**
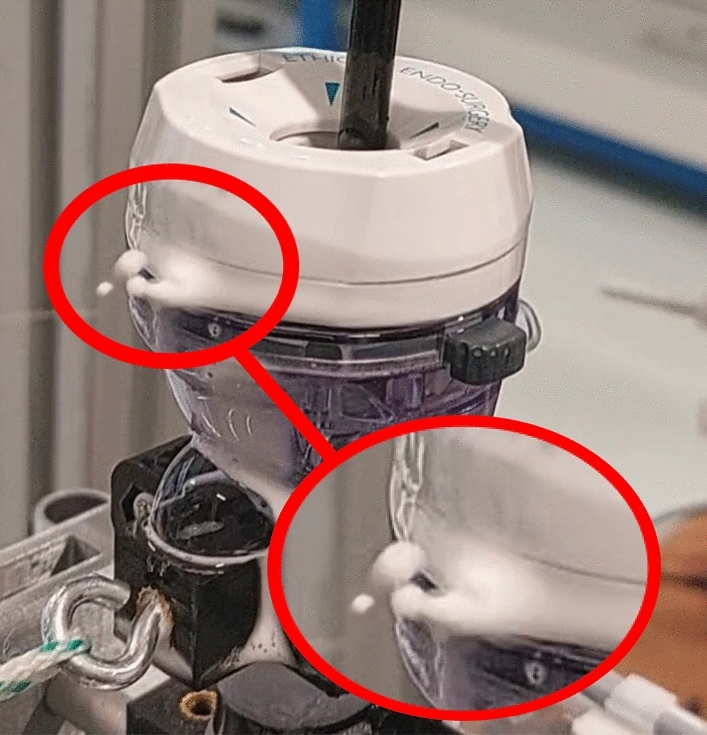
Expelled soap bubbles showing leakage between the head and body of trocar 17

Trocars of group 6 showed the highest leakage rates of all trocars and required further investigation. A soap test showed leakage from the connection between the head and body of the trocar (Fig. 8), a video of this test is provided in supplemental file 2.

The influence of the orientation of the connector orientation was tested by repeating step 8 of the protocol twice: first with the connectors were oriented perpendicular to the load and then with connectors being oriented collinear with the trocar load. In the collinear condition, leakage rates were 0.11 L/min and 0.13 L/min for the 5-mm and 10-mm instruments, respectively. In the perpendicular condition, leakage rates were 4.39 L/min with a 5-mm instrument and 5.42 L/min with a 10-mm instrument.

## Discussion

This study evaluated 25 individual trocars to find the influence of prolonged trocar manipulation on trocar leakage. After applying a series of manipulations on the trocars, it was found that for 20 out of 25 trocars, the leakage rates after manipulation did not differ from leakage rates before manipulation. Six trocars showed leakages larger than 0.25 L/min during one or more of the measurement steps. Two trocars showed damage caused by manipulation. Additionally, investigation of two trocars revealed undesirable leakage pathways.

### Trocar wear mechanisms and recommendations

The valve of trocar 1 developed a tear during loaded instrument manipulation. This trocar was designed such that the upper valve was mounted at the proximal end of the trocar. Therefore, forces applied to the instrument are directly transferred to the trocar valve, causing wear. In other trocars, the upper valve is located deeper in the trocar so that the instrument contacts the trocar body when the instrument is loaded. This configuration is recommended as it limits the friction force on the valve when the instrument is manipulated.

All 15-mm trocars of group 3 showed increased leakage after manipulation with a 5-mm instrument. Although inspection did not reveal any valve damage, the use of the 5-mm instrument in the 15-mm trocar potentially accelerated the wear of the valves. A 5-mm instrument in a 15-mm trocar is generally not recommended, although it could be required during specific procedures.

The reusable trocar in group 5 was tested three times, replacing the upper valve after every set. Despite the newly replaced valve, the second measurement with this trocar showed increased leakage, which could not be explained after inspection for damage. Results from this trocar group suggest that trocars can show varying leakage rates even within a specific trocar type. Robertson et al. [4] also reported significant variations between similar trocars.

Some trocar groups had leakage pathways while still in their new state, such as trocar group 6. These trocars leaked from a seal between the top and bottom part of the trocar, which could be caused by mechanical play in the connection. Trocars from groups 7 and 8 were of the same brand and similar design and did not show this behavior. A design or manufacturing flaw that can cause a very high increase in gas leakage that can cause a relevant clinical risk to the surgical staff should be detected within the Quality Assurance process of the manufacturer. It is unclear whether manufacturers’ testing and inspection protocols include any relevant loading conditions that simulate leakage during normal use of surgical instruments. These testing protocols could be included in the MDR for medical devices [31].

Trocar 24 developed wear on the lower trocar valve during instrument manipulation. While the instrument was inserted, the spring of the flap valve pushed the valve against the instrument shaft, causing wear on the valve and instrument. Loss of valve material prevented a tight seal which causes leakage. If the manufacturer performed a similar wear test as this study, this leakage could have been prevented by selecting more durable materials.

### Limitations and further research

The study was performed with trocars that were sent to the researchers by surgeon. This meant that group sample sizes were too small to provide a statistical comparison between different trocar groups. Furthermore, four individual trocars were tested and might not represent all trocars of the same type. Additionally, different instruments, than the ones used in this study, might cause more damage to trocar valves during instrument insertions.

Instrument manipulation, during laparoscopic surgery, consists of a combination of pivotal, axial, and radial displacements. Currently, studies presenting detailed information about the interacting force between laparoscopic instruments and trocars are lacking. Accurate force/torque measurements during surgical procedures would enable more accurate load cases in future experimental research.

Using a static trocar load in combination with axial manipulation was chosen for reproducibility, although it is a simplification of clinical practice. The setup resulted in loading a specific part of the trocar valve for a prolonged time, which is more severe than surgical practice. Despite this, leakage did not increase after manipulation. However, other peak loads that might occur in clinical practice might still cause damage to trocar valves.

The median leakage rates of empty trocars found in this study were in line with findings in previous research [16, 32, 33]. In general, most leakage measurements were below 0.1 L/min. However, this leakage would occur continuously during surgery. A surgical procedure of two hours would still result in 12 L of gas leakage per trocar with a leakage rate of 0.1 L/min potentially resulting in higher costs, more bottle changes, exposure to carcinogens [5] and, in case of outflow of particles, an increased risk to the surgical staff [34].

The leakages measured in this study are small compared to other leakages found in literature. For instance, opening the trocar stopcock results in more than four liters of leakage per minute [3], which is considered common practice during laparoscopic surgery [5, 35, 36]. During instrument insertions, leakage values up to 17 L/min and 31.3 L/min have been reported by Cahill et al. [3] and Robertson et al. [4], respectively.

Gas leakage through trocars is part of particle escape, along with leakage between the incision and the trocar and through instrument shafts. Several studies have quantified leakage through the trocar and the instrument shaft [3, 4, 16, 32, 37]. However, studies quantifying leakage between the incision and the trocars are still lacking. Therefore, future research should focus on this leakage mechanism and influencing factors.

The hazard of these gas leakages is caused by particles present in the abdomen that patients and staff could inhale. These risks can be mitigated by elements, such as OR ventilation and smoke evacuation systems. Modern operating theaters use air ventilation above 3000 m^3^/h [38]. Nevertheless, Hardy et al. stated that modern operating rooms could not prevent particle spread sufficiently [13]. Studies comparing different types and ventilation properties concerning particle exposure of the operating personnel are needed. The use of smoke removal systems during laparoscopy is an effective tool for removing smoke from the abdomen, improving visibility during surgery.

Whether these systems completely prevent particles from reaching the breathing space of operating room staff has not yet been studied. Therefore, until risks related to gas leakage are clarified, operating personnel should be aware of the potential hazard and adhere to current existing guidelines to mitigate exposure as much as possible.

## Conclusion

During this study, a protocol for prolonged instrument manipulation was developed and applied on 25 individual trocars. The results show that, for most trocars, prolonged instrument manipulation did not influence gas leakage rates in dynamic and static measurements. Nevertheless, the study did show that instrument manipulation caused damage to some trocars and revealed new unintended leakage pathways in individual trocars. These failure mechanisms, presented in this study, could guide new trocar development, design guidelines, and testing protocols and as a possible starting point for future research.

## Supplementary Information

Below is the link to the electronic supplementary material.

Supplementary file1 (MP4 18322 KB)

Supplementary file2 (MP4 37145 KB)
